# A case report of primary isolated extrahepatic hydatid cyst of the soft tissues of the breast and thigh

**DOI:** 10.1016/j.ijscr.2021.01.087

**Published:** 2021-01-29

**Authors:** Majid Samsami, Shohra Qaderi, Javad Zebarjadi Bagherpour, Don Eliseo Lucero-Prisno

**Affiliations:** a Department of General Surgery, School of Medicine, Shahid Beheshti University of Medical Sciences, Tehran, Iran; b School of Medicine, Shahid Beheshti University of Medical Sciences, Tehran, Iran; c Faculty of Management and Development Studies, University of the Philippines, Los Banos, Laguna, Philippines

**Keywords:** Hydatid cyst, Primary cyst, Extrahepatic, Breast, Thigh

## Abstract

**Introduction:**

Hydatid Disease (HD), or hydatidosis or echinococcosis, is an endemic infection and a major public health concern in the Mediterranean region. At times it involves the primary soft tissues, such as in the breast and muscle, though this is quite uncommon even in endemic areas.

**Presentation of cases:**

A. A 31 year-old woman complained of a gradual progressive, painless lump in the left axillary tail of spence for two years. Examination revealed a firm lump measuring 5cm × 5cm, non-mobile, in the left axillary tail of Spence. B. A 32 year-old woman presented with mild and continuous pain in lateral aspect of left thigh. On examination, there was a round, non-tender, non-mobile mass in the lateral aspect of her left thigh. Preoperative imaging studies in both patients revealed evidence of HD. Both of the patients underwent surgery and received Albendazole twice per day for 10 days, before and three months after surgery.

**Discussion:**

The disease can be diagnosed by serological and radiological modalities, both of which are not definitive. Ultrasonography should be the first diagnostic modality of soft tissue HD, however, MRI can be used to understand clearly the surgical involvements of structures. The standard treatment of soft tissue HD is surgery using pericystectomy techniques, as well as anthelmintic therapy.

**Conclusion:**

HD should be suggestive in soft tissues if mass is slowly developing and presenting with local extension, particularly in endemic countries. Excision of HD using pericystectomy technique is the first choice of intervention for HD of soft tissues.

## Highlights

• The hydatid cyst should be suggestive in soft tissues with slowly developing masses with local extension, particular in endemic countries.
• Excision of hydatid cyst in pericystectomy technique is the first choice of treatment for hydatid cyst of soft tissues.
• The standard treatment of muscular and breast echinococcusis is surgery as well as anthelmintic therapy.
• The diagnosis of HD should be excluded before any surgical excision or biopsy or Fine needle aspiration cytology.

## 1 Introduction

Hydatid Disease (HD), or hydatidosis or echinococcosis, is an endemic parasitic infection caused by the larval or cyst stage of the tapeworm Echinococcus granulosus (sch); and it is a major public health concern in the Mediterranean region [1]. This parasitic disease can affect any organ of the body, however, liver (55%–70%) followed by the lung (18%–35%) are the most commonly affected organs. The heart, brain, vertebral column, ovaries, pancreas, gallbladder, thyroid gland, breast, and bones have lower incidence rate of involvement. Unusual locations of HD account for about 8%–10% of cases [2,3]. Primary soft tissue involvement such as thigh and breast is uncommon even in endemic areas (0.5%–4.7%) [4]. HD of the breast is a very rare presentation, although it is challenging to differentiate it from other benign and malignant breast masses. Surgery still remains as the standard and main treatment for HD. To decrease the chance of anaphylaxis and tension of the cyst wall, to reduce the recurrence rate, and to sterilize the cyst, pre- and post-operative course of Albendazole and two weeks of Praziquantel should be considered [5]. Based on the Surgical CAse REport, 2020 (SCARE) guidelines [6] this paper presents two cases of primary isolated hydatid cyst in the breast and thigh, with a successful surgical cure.

## 2 Case presentations

### 2.1 Case 1

**Fig. 1:**
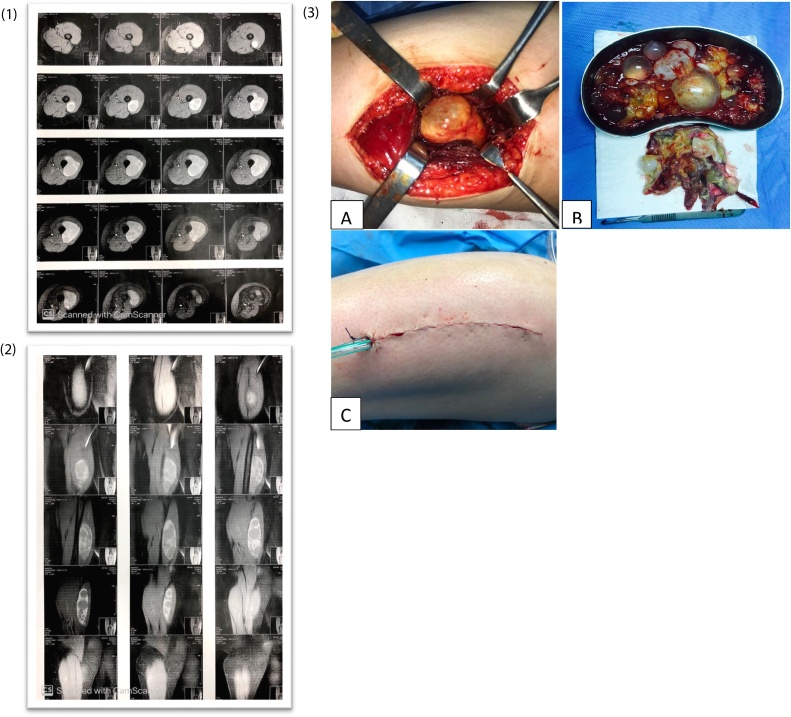
**1.** Ultrasonography of thigh showed 15 cm × 5 cm solid-cystic with multiple well defined cystic lesion. **2.** The MRI featured a large multisystem lesion. **3.** Photographs of lateral thigh show; A and B: Inner germinated layer of hydatid cyst. C: A drain fixed at the end of procedure.

A 32 year-old woman from a rural area, presented to the general surgery clinic with mild and continuous pain in the lateral aspect of her left thigh. Concomitantly, she was complaining of gradually increasing swelling in the lateral aspect of her left thigh for about 1 year duration. Family history and drug history was unremarkable. On examination, there was a round, non-tender, non-mobile mass in the lateral aspect of her left thigh. There was no overlying skin changes, ultrasound (US) showed 15cm × 5cm solid-cystic lesion in the lateral aspect of the left thigh with multiple well-defined cystic lesions. Magnetic Resonance Imaging (MRI) showed a relative large multisystem lesion in the lateral aspect of the thigh with connection to the femoral bone, in favor of hydatid cyst (Fig. 1**1** and **2**). Chest X-ray and abdominal ultrasound excluded lung and liver HDs. ELISA test of *Echinococcus Granulosa* titer test was negative. A primary muscular Hydatid cyst was diagnosed and a pericystectomy was chosen as the preferred surgical treatment. Treatment involved 400 mg of Albendazole twice per day for 10 days before and three months after surgery. The longitudinal incision (pericystectomy) was performed under spinal anesthesia in the anterolateral aspect of the left thigh (Fig. 1**3**). Cyst cavity was thoroughly irrigated with hypertonic saline and wound closed after putting a drain (Fig. 1**3**). The surgery has been carried out under the supervision of an attending general surgeon. Histopathological diagnosis confirmed the diagnosis of Hydatid Disease. The postoperative course of the patient was favorable. As Albendazole (400 mg twice per day) started 10 days before surgery, it was continued for three month after surgery and there was no evidence of recurrence of the lesion during the 8 month follow-up.

### 2.2 Case 2

**Fig. 2:**
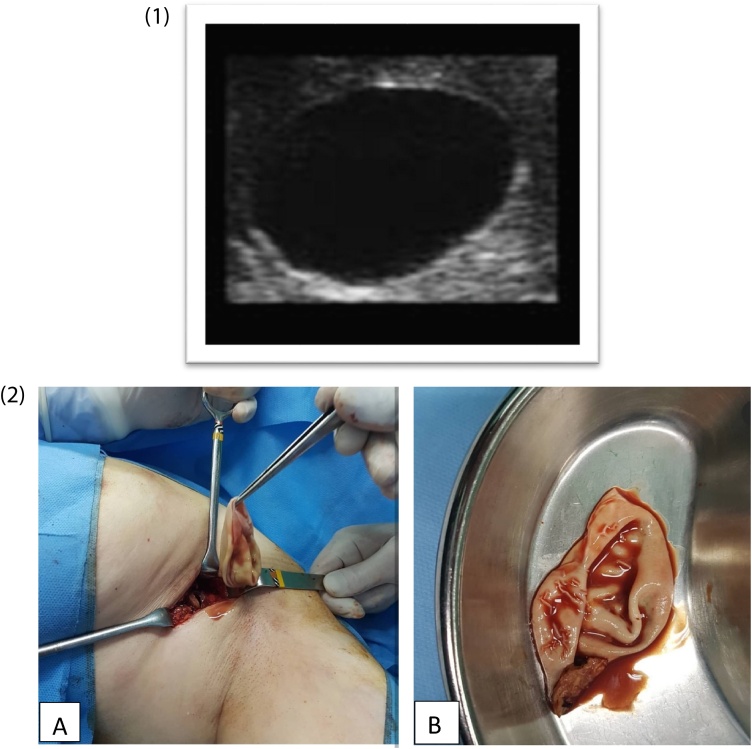
**1.** The US reveals a thick walled cystic lesion with floating membranes and internal echoes in the tail of left Spence. **2.** In photographs A and B the endcysts in excised breast mass confirmed the hydatid cyst.

A 31 year-old housewife presented with a gradually progressive, painless lump in the left axillary tail of Spence since two years. There was no history of injury, no discharge from the nipple, and no family history of breast cancer. She did not provide a history of close contact with any animal. Examination revealed a firm lump measuring 5cm × 5cm, non-mobile, in the left axillary tail of Spence. The rest of the left breast and nipple were normal. Collaborative ultrasonography study of the breasts revealed a thick walled cystic lesion with floating membranes and internal echoes in the region (Fig. 2**1**). The chest X-ray and ultrasonography of the abdomen of the patient were unremarkable. The patient underwent surgery for the removal of the lump. The surgery was performed by attending general surgeon and after general anesthesia a curvilinear incision in hair line margin, the cyst was removed in-toto. The excised cyst was oval in shape and measured 5 cm × 4.5 cm × 3 cm. When the cyst was opened, endocysts were found confirming it to be a hydatid cyst (Fig. 2**2**). Albendazole (400 mg twice per day) was continued and control imaging was performed.  There was no evidence of recurrence of the lesion during the 1 year follow-up.

## 3 Discussion

Hydatid Disease (HD) or hydatidosis or echinococcosis is an endemic disease and a major health concern in many parts of the world, including the Mediterranean region and the Middle East [1]. Iran is endemic for HD and has been reporting cases regularly [7]. Liver and lung are known as the most common affected organs in humans for HD [2]. Breast and muscle are known to be rare primary sites for HD even in endemic areas [4]. The breast and muscle HD may present as an indicator of a multisystem involvement or may indicate its occurrence in one part of the body [8]. The incidence rate of HD in the breast and muscle have been reported (0.27%–0.37%) and (1%–4%), respectively [9]. The high lactic acid level in the muscle tissues is considered unfavorable for the survival of the parasite [10]. In the current study, both of patients have not been previously operated for HD and investigation did not exhibit any hydatid cyst in any organs of their bodies. According to the endemicity of HD in Iran, these two cases were diagnosed having primary HD of the breast and muscle. The diagnosis of HD should be considered when a palpable, painless mass which gradually increases in size over months or years, in endemic areas [11,12]. It might mimic fibroademoma, phyllodes tumor, chronic abscess, or even malignancies, and this should be included in the differential diagnoses of breast lumps, especially in endemic regions [11,13]. The diagnosis of HD should be excluded before any surgical excision or biopsy or Fine needle aspiration cytology (FNAC), in order to avoid leakage of cyst components and accompanying risk of anaphylaxis and secondary hydatidosis. The disease can be diagnosed by serological and radiological tests, both of which are not definitive [14]. ELISA is 80%–100% sensitive and 88%–96% specific for hydatid of the liver but less sensitive for lung (55%–56%), or other organ involvement (25%–26%). Only positive hydatid serology is valuable, and negative serology does not exclude the diagnosis, as both of our cases had negative ELISA test for HD [15]. Ultrasonography should be the first diagnostic modality of soft tissues especially the breast. The sensitivity of ultrasonography is 95% and if vesicular fibrils are present its sensitivity increases to 100% [16,17]. However, hydatid cysts are usually present as uniloculated or multiloculated cystic lesions in ultrasonography; but this modality does not easily distinguish hydatid cyst from other simple or complicated breast masses [11]. The ultrasonography of both cases showed a multiloculated cystic lesion with internal septa and echogenic material that was suggestive of HD. Similar findings of ultrasonography have been reported by Kumar et al, Tavakoli et al. and Trabelsi et al. [11,18,19]. MRI findings are non-specific, but may be helpful in the distinct identification of involved structures for surgical planning [17]. MRI usually reveals a capsular enhancement which is often seen in an infected cyst; and a hydatid cyst may present as a cyst lesion with no evidence of capsular enhancement, which a breast abscesses may indicate a similar finding. MRI was used to make the structures in both of the patients distinct for the surgical procedure. Mammography usually demonstrates a hydatid cyst as a well circumscribed mass of variable density, and may reveal ring shaped structures of internal bands within the cyst [13,17]. The standard treatment of muscular and breast HD is surgery as well as anthelmintic therapy. The first technique of choice is pericystectomy, that is removing the entire cyst without breaking the wall in an intraoperative field soaked with 3% saline solution (as precautionary measure), [19,20]. The excision of hydatid cyst in the muscle can sometimes be problematic, due to the absence of cleavage lines, especially when the cyst is infected. The adherence of the cyst to the blood vessels and nerves makes it particularly tight making excision difficult [21]. Some procedures such as percutaneous techniques using puncture, aspiration, alcohol or sclerosing product injection and re-aspiration, or percutaneous drainage with injection but no re-aspiration, are well-validated for eruptive to surgical excision among specific patients [20,22]. Pre-surgical and post-surgical anthelmintic drug therapy have been shown to decrease the incidence rate of recurrence [23]. Recurrent HD in the breast and muscle after surgery is rare. Both cases underwent anthelmintic drug therapy for 3 months after surgery, and recurrence of hydatid cyst was not seen after 4 months of follow up.

## 4 Conclusion

Hydatid Disease should be suggestive in soft tissues presenting with slowly developing masses with local extension, particularly in endemic countries. MRI is the most useful diagnostic modality of HD of the soft tissues and for planning the surgery, whenever ultrasonography or serological tests do not sufficiently characterize the disease. Excision of the hydatid cyst using pericystectomy technique is the first choice of treatment for HD of the soft tissues.

## Conflicts of interest

There is no conflicts of interest.

## Funding

None.

## Ethical approval

This is a case report paper.

## Consent

Informed consent was obtained from the both of the patients, for publication of this case report and accompanying images. A copy of the written consent is available for review by the Editor-in-Chief of this journal on request.

## Author contribution

MS and JZB conceptualized the study, Sh.Q acquisition of data, and drafting the manuscript, D.E.L.P revising for critical intellectual concept and approved of the version to be submit.

## Registration of research studies

Not applicable.

## Guarantor

Corresponding author is Dr. Javad Zebarjadi Bagherpour accept full responsibility for the work and/or the conduct of the study, had access to the data, and controlled the decision to publish.

## Provenance and peer review

Not commissioned, externally peer-reviewed.
